# Flavonoids in Plants and Human Health: From Biosynthesis to Neurodevelopmental and Neurodegenerative Disorders

**DOI:** 10.3390/molecules31010066

**Published:** 2025-12-24

**Authors:** Joanna Lemanowicz, Kinga Gawlińska, Iwona Jaskulska, Dariusz Jaskulski, Mateusz Sar

**Affiliations:** 1 Division of Biochemistry, Faculty of Medicine, Bydgoszcz University of Science and Technology, Bernardyńska 6 St., 85-029 Bydgoszcz, Poland; 2 Department of Clinical Pharmacy, Medical College, Jagiellonian University, Medyczna 9, PL, 30-688 Krakow, Poland; kinga.gawlinska@uj.edu.pl (K.G.); mateusz.sar@student.uj.edu.pl (M.S.); 3 Department of Agronomy and Food Processing, Faculty of Agriculture and Biotechnology, Bydgoszcz University of Science and Technology in Bydgoszcz, 7 Kaliskiego St., 85-796 Bydgoszcz, Poland; iwona.jaskulska@pbs.edu.pl (I.J.); dariusz.jaskulski@pbs.edu.pl (D.J.)

**Keywords:** antioxidant activity, plant secondary metabolites, health-promoting effects, antioxidant properties

## Abstract

Flavonoids are a class of natural plant compounds that are categorised within the polyphenolic group. It is widely acknowledged that their structural diversity results in a wide distribution within food sources, thus leading to a concomitant wide spectrum of biological activity. This review provides an updated overview of the main flavonoid subclasses, including flavonols, flavones, flavanones, flavanols, anthocyanins, and isoflavones, and includes an examination of their chemical properties and biosynthetic pathways. The present study will discuss the influence of biotic and abiotic factors on flavonoid function in plants, including their role in ultraviolet protection, stress tolerance, and defence signalling. The regular consumption of foods rich in flavonoids has been demonstrated to be associated with a reduced risk of chronic diseases, including cardiovascular, metabolic diseases, neurodegenerative and neurodevelopmental disorders. This observation underscores the significance of flavonoids in a balanced diet. Medicinal plants play an important role in this task. The mechanisms of action of this substance include antioxidant and anti-inflammatory effects, modulation of signalling pathways, and neuroprotective functions. The present findings underscore the significance of flavonoids as multifunctional bioactive molecules, which hold considerable potential for preventive and therapeutic applications. However, further well-designed human studies are necessary to determine effective dosage, long-term safety, and clinical relevance.

## 1. Introduction

In recent years, flavonoids—natural plant-derived compounds belonging to the polyphenol class—have attracted considerable attention in biochemical and clinical research [1,2,3]. The primary biological role of these compounds in plants is to contribute to flower pigmentation, thereby enhancing visual attraction for pollinators [4]. Flavonoids occur as pigments dissolved in cell sap, imparting a yellow hue to plant tissues, particularly flowers and leaves. They are less commonly found in fruits, bark, and wood, while their occurrence in seeds is rare.

**Figure 1:**
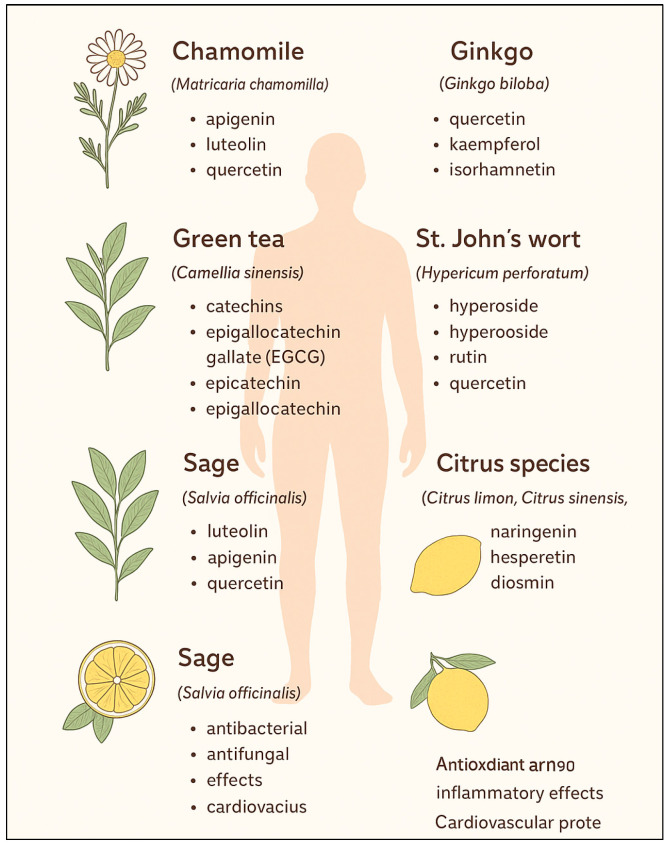
Medicinal plants—Flavonoids—Health effects.

Phytochemical studies indicate that a high level of flavonoids correlates with antioxidant activity and the potential pharmacological properties of herbal raw materials. Numerous flavonoids are commonly identified in medicinal plants, including quercetin, which occurs, among others, in stinging nettle leaves (*Urtica dioica*) and hawthorn flowers (*Crataegus* spp.). Other important representatives of this group include luteolin and apigenin, which are abundantly present in vervain (*Verbena officinalis*) and German chamomile (*Matricaria chamomilla*). Chinese tea (*Camellia sinensis* L.) is a natural source of catechin. Moreover, many herbal materials, such as ginkgo (*Ginkgo biloba*), contain flavonol glycosides, including rutin and isoquercitrin, which are characterized by high antioxidant activity [3] (Figure 1).

Okoye et al. [5] demonstrated that, in addition to fruits and vegetables, several bacterial species (*E. coli*, *C. glutamicum*, *L. lactis*, and *Bacillus* sp.) are capable of synthesizing flavonoids from plant biomass.

Due to their broad spectrum of beneficial biochemical effects, flavonoids have wide applications in human nutrition and health [6]. These compounds, classified as low-molecular-weight secondary plant metabolites, exhibit numerous health-promoting properties, most notably antioxidant and anti-inflammatory activities [7].

The growing prevalence of infectious and non-communicable diseases currently represents one of the major threats to global public health. The COVID-19 pandemic has highlighted the worldwide consequences of these challenges, while also underscoring the complexity and length of the drug discovery and development process.

Flavonoids, known since antiquity as plant pigments, were not structurally characterized until the late nineteenth century. In 1930, Albert Szent-Györgyi isolated a novel substance from citrus fruits, which he termed “vitamin P.” Subsequent studies revealed, however, that “vitamin P” was not a single compound but a mixture of flavonoids, with rutin (rutoside) identified as the major component. Today, it is well established that flavonoids are not vitamins in the strict biochemical sense but rather constitute a large group of polyphenolic compounds [8,9].

By the late 1990s, accumulating epidemiological evidence supported the cardioprotective role of dietary flavonoids. One example was the observation of their hypolipidemic effects in individuals with hypercholesterolemia consuming black tea [10]. To date, more than 9000 flavonoid compounds have been identified [11], which are classified into distinct subgroups based on their chemical structure [12,13].

**Figure 2:**
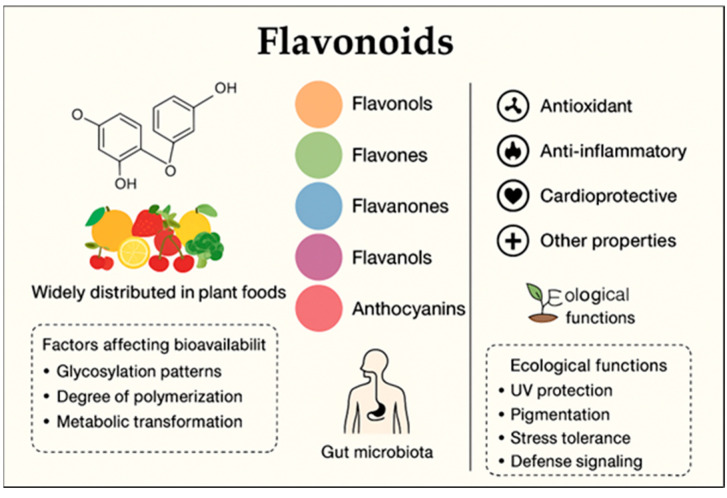
Bioactivity of flavonoids.

Flavonoids are compounds with multifaceted biological activity, exhibiting antimicrobial, anti-aging, cardio- and radioprotective, anti-inflammatory, and antidiabetic properties, which makes them promising candidates for research into disease prevention and therapy (Figure 2) [14,15].

## 2. Biological Activity of Flavonoids

Flavonoids are a broad class of phenolic compounds that are stored in the vacuoles of plant cells and occur in virtually all parts of higher plants. They can be found in fruits, vegetables, herbs, seeds, and flowers, for example in fruits and berries (apples, blueberries, lemons), vegetables (onions, cabbage, carrots), legumes (beans, peas, lentils), seeds and nuts (soybeans, almonds), herbs and spices (mint, sage), as well as plant-based beverages and their derivatives (green tea, cocoa) [16].

In plants, flavonoids may occur in two forms: as free aglycones or as *β*-glycosides containing sugar residues. A general characteristic of flavonoids is their good solubility in alkaline solutions, in which they develop a yellow coloration. Aglycones are weakly polar and therefore readily soluble in organic solvents. In contrast, flavonoids in the form of glycosides are soluble in water and ethanol but insoluble in ether, benzene, and chloroform [17,18].

**Figure 3:**
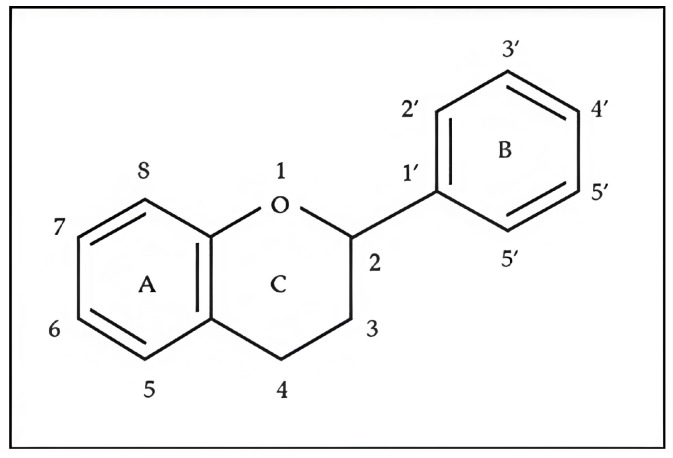
Primary structural skeleton of flavonoids.

The basic flavonoid skeleton consists of 15 carbon atoms arranged in a flavan framework (C6–C3–C6), composed of two benzene rings (A and B) linked by a heterocyclic pyran or pyrone ring (C) (Figure 3).

Flavonoids are classified into various subtypes depending on the position of the B ring relative to the C ring, as well as the degree of oxidation and unsaturation of the C ring. Based on these and other structural features, flavonoids are divided into fourteen structural classes [3,16] (Table 1).

The structural heterogeneity of flavonoids strongly determines their bioactivity and pharmacokinetics. The bioavailability of dietary flavonoids shows substantial variability, arising from differences in conjugation, polarity, and interactions with the gut microbiota [19].

Flavonoids exert a broad range of health-promoting effects through multiple mechanisms, primarily due to their potent antioxidant activity and their ability to modulate various enzymes [3,20]. They chelate metal ions such as Zn^2+^, influencing oxidative processes, including those associated with oxidative stress [21]. By scavenging reactive oxygen and nitrogen species (ROS and RNS), flavonoids protect cells against oxidative injury. They may also regenerate other antioxidants, including vitamins C and E, and inhibit lipid peroxidation [22]. Many flavonoids suppress inflammatory signaling pathways (e.g., NF-κB), reduce COX-2 expression, attenuate the production of pro-inflammatory cytokines, and enhance antioxidant defense systems, collectively lowering tissue inflammation [23].

Dietary flavonoids, such as flavonols, improve endothelial function and reduce vascular oxidative stress and inflammation, correlating with a decreased risk of cardiovascular diseases. Several compounds exhibit veno- and vasoprotective properties by improving vascular flow, reducing congestion and edema, and stabilizing endothelial and extracellular matrix integrity. These effects support their use as adjuvant agents in the prevention and management of venous insufficiency, bruising, bleeding tendencies, and, more broadly, as supportive therapy in atherosclerosis due to endothelial protection [24,25]. Additional metabolic benefits, such as improved glycemic control, have also been reported [20].

Wang et al. [26] showed that several flavonoids reduce arachidonic acid production and suppress phospholipase A2, cyclooxygenase, and NOS activities, limiting the synthesis of inflammatory mediators, including prostaglandins, leukotrienes, and NO. Flavonoids can act as hydrogen donors to α-tocopherol radicals, delaying the oxidation of low-density lipoproteins (LDL) by interacting with α-tocopherol radicals [27].

Selected flavonoids induce apoptosis, inhibit proliferation, block angiogenesis, and modulate cancer-related signaling pathways (e.g., NF-κB, MAPK), providing potential for chemoprevention or adjuvant cancer therapy [28]. They also modulate numerous enzymes either through direct inhibition or regulation of gene expression. Examples include inhibitors of protein kinases, oxidoreductases [29], enzymes involved in hormone metabolism, and two key digestive enzymes—α-glucosidase and α-amylase [30].

Many flavonoids downregulate inflammation-associated pathways such as NF-κB and MAPK, decreasing the expression of pro-inflammatory cytokines (e.g., TNF-α, IL-6) and enzymes (e.g., COX-2, iNOS) [31]. In addition, flavonoids stabilize mitochondrial function, reduce oxidative stress-induced apoptosis, and modulate pathways associated with cell proliferation and differentiation [32].

**Figure 4:**
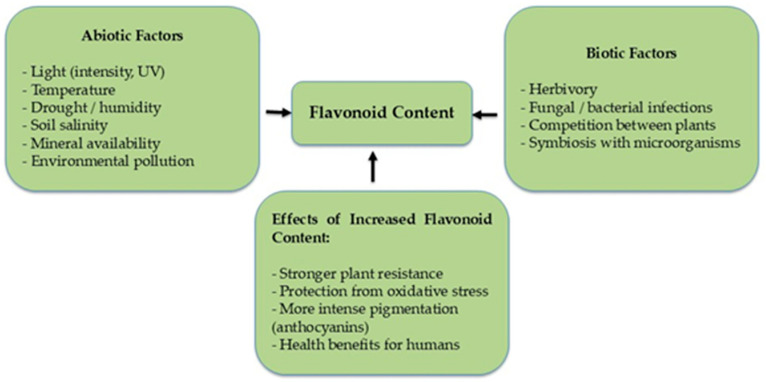
Influence of biotic and abiotic factors on flavonoid content in plant.

As phytoalexins, flavonoids protect plants against damage induced by environmental and biological stressors, including UV (ultraviolet) radiation, low temperature, pathogens, and insect herbivory [33,34]. The biosynthesis of flavonoids is strongly regulated by environmental factors that influence gene expression and the activity of enzymes involved in phenolic metabolism. Because flavonoids play protective roles in plants, their levels often increase in response to stress (Figure 4) [35].

Light intensity and quality, particularly UV-B radiation, are major regulators of flavonoid biosynthesis [36]. UV-B induces the expression of genes such as CHS (chalcone synthase) and CHI (chalcone isomerase), leading to increased accumulation of flavonoids, especially anthocyanins and flavonols. This response enhances hydroxylation levels and strengthens the antioxidant capacity of plants. Both low and high temperatures can modulate flavonoid biosynthesis. Thermal stress frequently increases phenolic compound content, which enhances antioxidant protection against oxidative damage [37].

Under drought stress, plants accumulate anthocyanins to protect tissues from excessive sunlight, reduce transpiration, and decrease stomatal density, thereby preventing water loss [38]. Research by Schultz et al. [39] demonstrated that flavones, flavonols, flavanols, and anthocyanins exhibit high tolerance to cold stress. Water deficit generally enhances flavonoid biosynthesis—particularly anthocyanins and flavonols—which mitigate oxidative stress caused by the accumulation of reactive oxygen species (ROS) [40].

Pathogen attack or mechanical tissue injury induces plant defense responses in which flavonoids play a central role as phytoalexins and antioxidants [41]. Exposure to heavy metals (e.g., Cd, Pb, Cu) and ozone can also elevate flavonoid levels, as these compounds contribute to the neutralization of free radicals generated under oxidative stress [42]. Pathogen infection induces the expression of genes encoding key enzymes of the flavonoid biosynthetic pathway, such as CHS and CHI [43].

Some plants secrete flavonoids and other phenolic compounds into the environment, influencing the growth of neighboring species (allelopathic effect). In response, plants may increase their own flavonoid biosynthesis to protect themselves against the toxic impact of allelochemicals [40].

The biosynthesis of flavonoids in plants proceeds through two key metabolic pathways: the shikimate (phenylpropanoid) pathway, which generates the C6–C3 backbone, and the acetate (polyketide) pathway, which provides the C2 units required for forming polymeric structures l, 2 [44,45]. Enzymes involved in flavonoid biosynthesis belong primarily to oxidoreductases, transferases, lyases, and ligases [46]. The predominance of oxidoreductases underscores the central role of redox reactions in shaping this metabolic route [35]. Examples of enzymes catalyzing and regulating flavonoid formation include PAL, which controls the flux through the phenylpropanoid pathway; IFS, the key enzyme in isoflavone biosynthesis; FSN I and FSN II, two synthases responsible for flavone formation; and LAR and ANR, reductases involved in the synthesis of flavan-3-ols, the precursors of proanthocyanidins (Table 2) [35,47,48].

**Figure 5:**
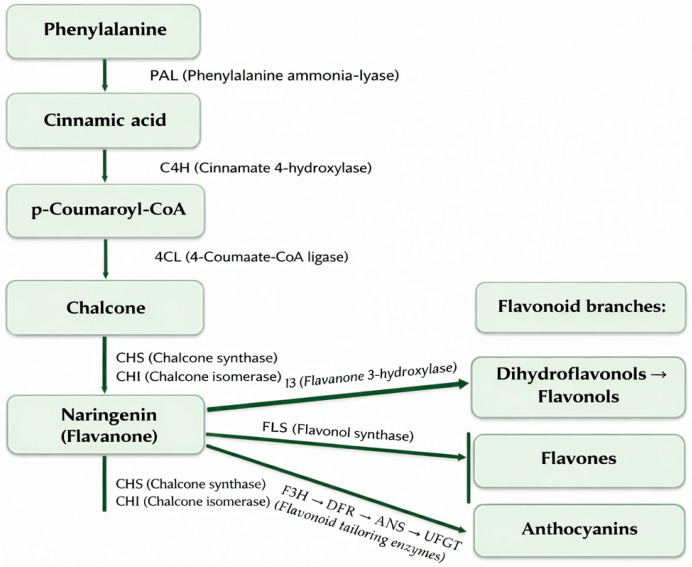
Flavonoid biosynthesis.

The shikimate pathway leads to the formation of aromatic amino acids such as phenylalanine, a crucial precursor for flavonoid biosynthesis. Phenylalanine undergoes deamination catalyzed by phenylalanine ammonia-lyase (PAL), forming cinnamic acid, which is subsequently hydroxylated by cinnamate 4-hydroxylase (C_4_H) to p-coumaric acid. Activation of this compound yields p-coumaroyl-CoA, which then condenses with three malonyl-CoA units in a reaction catalyzed by chalcone synthase (CHS). This process produces a compound with the characteristic C6–C3–C6 flavonoid skeleton. Chalcone is then isomerized by chalcone isomerase (CHI) to form a flavanone, which serves as the central intermediate for subsequent modifications—including hydroxylation, methylation, and glycosylation—ultimately generating diverse flavonoid classes such as flavonols, isoflavones, and anthocyanins (Figure 5) [47,49].

The acetyl–malonyl pathway cooperates with the phenylpropanoid pathway by supplying malonyl-CoA units that condense with p-coumaroyl-CoA through the action of CHS to form chalcone. In this pathway, polyketide synthases (PKS) play a key role, followed by CHI, which catalyzes the isomerization of chalcone to flavanone. Further enzymatic modifications include hydroxylation mediated by flavanone-3-hydroxylase (F3H), methylation by O-methyltransferases, and glycosylation catalyzed by UDP-glycosyltransferases (UGTs). Together, these pathways, through a series of enzymatic modification reactions, enable plants to produce a wide spectrum of flavonoids and generate structurally diverse phenolic compounds with varied biological functions [47,49].

## 3. Flavonoids in Food and Nutrition

Flavonoids constitute a major group of bioactive compounds present in plant-based foods, playing a vital role in human nutrition and health maintenance. Consumption of flavonoids from fruits, vegetables, tea, cocoa, and herbs has been linked to numerous health benefits, including antioxidant, anti-inflammatory, and cardioprotective effects [1,50]. In recent years, interest in nutraceuticals and functional foods enriched with flavonoids has surged, driven by their potential to prevent chronic diseases and support healthy aging [51,52]. This trend reflects two key factors: increasing consumer demand for health-promoting foods and a growing body of evidence associating dietary flavonoids with positive health outcomes [53,54]. In recent years, medicinal plants have gained considerable attention due to their potential impact on human health and their richness in bioactive secondary metabolites [50]. The flavonoids identified in these plant materials exhibit a broad spectrum of biological activities, which may influence the progression of various diseases through antioxidant, anti-inflammatory, and anti-atherosclerotic mechanisms [50]. In the present study, particular emphasis was placed on flavonols such as quercetin and rutin, which are widely recognized for their capacity to reduce oxidative stress and attenuate inflammatory responses, both of which are critically involved in the pathogenesis of cardiovascular and neurodegenerative disorders. Additionally, luteolin and apigenin—abundant in *Matricaria chamomilla* and *Verbena officinalis*—demonstrated immunomodulatory properties and inhibitory effects on cell proliferation, suggesting their potential relevance in the context of inflammatory conditions and selected malignancies [52].

### 3.1. Flavonoid Content in Foods and Dietary Intake

Flavonoid content in plant foods exhibits considerable variability, influenced by botanical species, genetic factors, and environmental conditions. Several factors affect flavonoid biosynthesis and accumulation, including cultivar, soil composition, light exposure, temperature, and water availability [55,56]. Moreover, the stage of maturity at harvest strongly influences the levels of anthocyanidins, flavanones, and flavonols in fruits, vegetables, and medicin plant [38].

Flavonoid subclasses are unevenly distributed across plant-based foods, with certain foods containing particularly high concentrations of specific compounds. Anthocyanidins, responsible for the characteristic red, blue, and purple colors in berries such as blueberries and strawberries, are predominantly found in these fruits [56,57,58]. Flavan-3-ols, including catechins and epicatechins, are abundant in tea and cocoa, contributing to their antioxidant and cardioprotective activities [52,55]. Citrus fruits are the primary source of flavanones, whereas onions, broccoli, and herbs such as parsley are rich in flavonols and flavones [56,58].

Flavonoid intake varies significantly across populations, reflecting differences in dietary habits, food availability, and cultural preferences. Daily intake ranges widely, with higher levels typically observed in regions consuming diets rich in fruits, vegetables, tea, and cocoa. Overall dietary patterns strongly influence flavonoid intake: plant-based diets, including the Mediterranean diet and the Dietary Approaches to Stop Hypertension (DASH), are associated with higher flavonoid consumption compared to diets richer in animal-based foods [19,53]. Regular consumption of berries, citrus fruits, tea, cocoa, onions, and cruciferous vegetables contributes substantially to total daily flavonoid intake, emphasizing these foods as key sources of bioactive compounds. Given the diversity and uneven distribution of flavonoid subclasses, sufficient intake requires frequent and varied consumption of flavonoid-rich foods. Including a wide range of fruits, vegetables, herbs, and plant-based beverages ensures exposure to multiple flavonoid subclasses and maximizes potential health benefits [49,55] (Table 3).

### 3.2. Bioavailability, Processing, and Storage

The nutritional effects of dietary flavonoids depend not only on their content in foods but also on their bioavailability, which determines the extent to which these compounds are absorbed, metabolized, and reach target tissues [59]. Following ingestion, flavonoids undergo extensive metabolism in the gastrointestinal tract, where the gut microbiota plays a crucial role in breaking down complex flavonoid structures into smaller, absorbable metabolites [60]. Once absorbed, flavonoids circulate primarily as conjugated metabolites, including glucuronides, sulfates, and methylated derivatives, which are considered the active forms mediating their health benefits [59,61].

Several dietary and processing factors influence flavonoid bioavailability. Dietary fat enhances the absorption of lipophilic flavonoids [62], while fermentation processes, as in tea or cocoa production, increase the proportion of bioactive metabolites [56,57]. Physical processing methods, such as milling, pureeing, or juicing, facilitate the release of flavonoids from plant matrices. Conversely, high temperatures, prolonged exposure to oxygen or light, and extended storage can degrade flavonoids or alter their chemical structures, reducing their physiological activity [56,63]. Understanding these factors is crucial for optimizing the health-promoting effects of flavonoid-rich foods, highlighting the importance of both suitable dietary sources and optimal preparation and storage methods.

Flavonoid content and bioavailability are significantly influenced by food processing and storage techniques. Cooking methods such as boiling, steaming, baking, and frying can cause substantial flavonoid losses due to thermal degradation and leaching into cooking water [64,65]. However, in some cases, these processes may enhance bioavailability by disrupting plant cell walls and the food matrix, liberating bound compounds [57,66]. Fermentation and enzymatic transformations also play a key role in modifying flavonoid profiles, increasing the concentration of absorbable bioactive metabolites. Industrial preservation methods, such as pasteurization, freezing, drying, and canning, show variable effects: some may partially degrade flavonoids, whereas others preserve them by minimizing exposure to oxygen, light, and degradative enzymes [66]. Knowledge of these effects is critical for consumers and the food industry to maximize flavonoid retention and bioavailability.

### 3.3. Health Effects of Flavonoids

Regular consumption of flavonoid-rich foods has been associated with a reduced risk of several chronic diseases, reinforcing their nutritional relevance in a balanced diet. Epidemiological and interventional studies indicate that diets high in flavonoids may contribute to the primary prevention of cardiovascular diseases (CVDs) through improvements in endothelial function, blood pressure regulation, and lipid profiles [53,54]. Evidence also supports a protective role for flavonoids in neurodegenerative disorders, including cognitive decline and Alzheimer’s disease, via antioxidant and anti-inflammatory mechanisms [49,67]. Additionally, flavonoid intake has been linked to reduced risk of type 2 diabetes, potentially through enhanced insulin sensitivity, and may contribute to cancer prevention by modulating cell proliferation, apoptosis, and inflammatory pathways [49,53,56] (Table 4).

Flavonoids represent a class of significant bioactive compounds present in plant-based foods, with a demonstrated role in promoting cardiovascular, neuroprotective, and metabolic health. It is imperative that a wide variety of flavonoid-rich foods is consumed on a regular basis to ensure optimal health benefits. Further research is required to provide greater clarity on the bioavailability of these substances and their interactions with gut microbiota.

## 4. Flavonoids in the Prevention and Treatment of Neurodevelopmental and Neurodegenerative Disorders

In neurodevelopmental disorders (e.g., autism spectrum disorder, ASD; attention deficit hyperactivity disorder, ADHD) and neurodegenerative disorders (e.g., Alzheimer’s disease, Parkinson’s disease), mechanisms such as oxidative stress, chronic inflammation, synaptic dysfunction, and the gut-brain axis play an important role [68,69]. The multi-directionality of the phenomenon is confirmed by the neuroprotective components identified in MFH plants from the Leguminosae family, which reduce oxidative stress, attenuate neuroinflammation, inhibit apoptosis and improve mitochondrial function, confirming the integrated action of flavonoids and related bioactive substances on neuronal survival pathways [70]. Numerous flavonoids penetrate the blood-brain barrier and accumulate in the central nervous system and have been shown to possess antioxidant, anti-inflammatory, and neuroprotective properties, suggesting their capacity to modulate these processes. Recent studies have demonstrated that the regular consumption of foodstuffs rich in flavonoids can effectively enhance cognitive capabilities in humans by a positive effect on the cerebrovascular system, which can improve cognitive performance by increasing blood flow and stimulating neurogenesis in the brain [71]. Flavonoids have been demonstrated to exert a neuroprotective effect by maintaining the appropriate quality and number of neurons in key brain areas, thereby preventing the occurrence and development of diseases responsible for cognitive decline [72]. This indicates their potential for use in prevention and adjunctive therapy.

### 4.1. Oxidative Stress and Mitochondrial Dysfunction

Oxidative stress, resulting from an overproduction of reactive oxygen species (ROS), and mitochondrial dysfunction are common features of numerous neurodevelopmental and neurodegenerative diseases. Increased markers of lipid peroxidation and decreased activity of antioxidant enzymes have been reported in ASD and ADHD. In Alzheimer’s disease, ROS contribute to neuronal damage and *β*-amyloid aggregation. The limited availability of effective treatments capable of halting or reversing disease progression has increased interest in natural compounds with neuroprotective potential, particularly flavonoids [73]. Flavonoids derived from medicinal plants have been shown to mitigate oxidative stress and preserve mitochondrial function, thereby protecting cells against mitochondriopathies and associated pathologies [74]. The Nrf2 signaling pathway plays a central role in cellular defense against oxidative stress by increasing the expression of antioxidant proteins and detoxification enzymes. Disruption of this pathway has been linked to result in mitochondrial dysfunction and impaired immune system, thereby increasing neuroinflammation and accelerating the progression of brain disorders [75]. Oxidative stress-induced apoptosis has been identified as a key factor in the pathophysiology of Parkinson’s and Huntington’s disease. Excessive ROS production leads to mitochondrial impairment and progressive neuronal loss in specific brain areas, including the cerebral cortex, substantia nigra, and hippocampus [75]. Neuroprotective flavonoids, such as luteolin, quercetin, hesperetin, and epicatechin, have been shown to activate the Nrf2 pathway, thereby enhancing the expression of antioxidant genes and protecting neurons from oxidative stress. These effects have been demonstrated in both in vitro and in vivo studies [75,76,77]. Such compounds offer promising avenues for mitigating oxidative damage and preserving mitochondrial function, potentially slowing the progression of neurodegenerative and neurodevelopmental disorders.

### 4.2. Neuroinflammatory Cytokines and Immune Modulation

Chronic brain inflammation, characterized by microglial activation and the production of proinflammatory cytokines such as IL-6, TNF-α, IL-1β, has been observed in both neurodevelopmental and neurodegenerative disorders. Flavonoids have been shown to inhibit the NF-κB pathway, thereby regulating the expression of inflammatory genes. Additionally, flavonoids can modulate MAPK and JAK/STAT signaling pathways [78]. Preclinical studies have demonstrated that luteolin reduces the secretion of proinflammatory cytokines, including IL-1β, TNF-α, and IL-6 [30,31,32]. Moreover, luteolin inhibits the phosphorylation of p65 and p38 in LPS-stimulated murine C6 cells [79]. Similarly, baicalein modulates microglial polarization by decreasing proinflammatory markers (TNF-α, iNOS, IL-1β, IL-6, CD16, CD86) and increasing anti-inflammatory markers (Arg-1, CD206) through STAT1 activation and TLR4/NF-κB inhibition in LPS/IFN-γ-stimulated BV2 cells (source to be added). In animal models of ASD, luteolin has been shown to reduce cytokine levels while improving social behavior and cognitive function [80]. Quercetin has also been observed to decrease IL-13 and TNF-α levels and ameliorate autism-like behaviors in rat models [81].

### 4.3. Gut-Brain Axis in Neurodevelopmental and Neurodegenerative Diseases

The microorganisms in the human body are crucial for gut and brain function, forming the microbiota-gut-brain axis. This axis is increasingly recognized in research on psychiatric, neurodevelopmental, age-related, and neurodegenerative disorders. Communication occurs via the immune system, tryptophan metabolism, the vagus nerve, and the enteric nervous system, involving microbial metabolites like short-chain fatty acids (SCFA), branched-chain amino acids, and peptidoglycans [82].

The influence of gut microbiota on brain function is characterized by its capacity to modulate inflammation and short-chain metabolites. Flavonoids have been demonstrated to modulate microbiota composition and to exert an indirect influence on the gut-brain axis, a factor which is of particular significance in the context of neurodevelopmental and neurodegenerative symptoms. A multitude studies have demonstrated that flavonoids possess the capacity to regulate the growth of specific bacterial taxa, in addition to altering the structure and function of gut microbiota, which contributes to a wide range of health benefits [83]. Flavonoids from medicinal plants may modulate the gut–brain axis by influencing gut microbiota composition and reducing oxidative stress, thereby potentially mitigating neuroinflammatory processes associated with neurodegenerative diseases [78]. Zhang and Park [84] demonstrated positive responses of *Vitex trifolia*, *Plantago major*, *Apocyni Veneti Folium*, and *Eucommiae folium* in alleviating both neuronal and intestinal inflammation.

In preclinical models of ASD, flavonoids such as luteolin and quercetin have been demonstrated to reduce neuroinflammation, oxidative stress, and intestinal permeability, while concomitantly enhancing social and cognitive behaviors. These effects are associated with modulation of gut function and microbial metabolites, including SCFA [6,85]. Despite the lack of clinical evidence in human subjects, the findings support the hypothesis that flavonoids have a potential role in modulating the gut–brain axis in neurodevelopmental disorders. In models of Alzheimer’s disease, luteolin and kaempferol have been shown to reduce neuroinflammation and oxidative stress, improve cognitive function, and be associated with favourable changes in the composition of the gut microbiome. The benefits of flavonoids have been corroborated by clinical and epidemiological studies: higher, regular flavonoid consumption has been associated with slower age-related cognitive decline and better cognitive health in older adults [86,87]. Many herbal and medicinal plants, such as garlic, ginger, sage, and turmeric, help prevent Alzheimer’s disease due to the presence of flavonoids in them [88,89,90,91]. Studies conducted by Jin et al. [92] indicate that baicalin isolated from *Scutellaria baicalensis* Georgi (Huang Qin) reduces microglia-mediated neuroinflammatory responses in the brains of mice with Alzheimer’s disease (Table 5).

Flavonoids, on the other hand, are typically well tolerated via dietary consumption, and the occurrence of adverse effects is minimal. However, high doses have been associated with mild gastrointestinal complaints, headaches, and potential drug interactions due to modulation of cytochrome P450 enzymes. Flavonoids are classified as low-risk compounds (LD50 > 2000 mg/kg), although some studies indicate that excessive consumption or supplementation may have genotoxic, endocrine, or prooxidant effects [94,95,96].

Despite the previous descriptions of the various mechanisms involved in flavonoid-mediated neuroprotection, growing evidence suggests that flavonoids do not act through individual pathways, but rather as multi-target modulators of interconnected signaling networks (Table 5). This characteristic is of relevance in complex neurodevelopmental and neurodegenerative disorders, in which oxidative stress, neuroinflammation, mitochondrial dysfunction, synaptic impairment, and gut dysbiosis coexist and mutually reinforce each other.

## 5. Conclusions

Flavonoids are a class of natural polyphenols, which have been shown to possess antioxidant, anti-inflammatory, and neuroprotective properties. These substances have been demonstrated to modulate the gut–brain axis, traverse the blood-brain barrier, and support cognitive function. Flavonoids from medicinal and herbal plants exhibit antioxidant, anti-inflammatory, and neuroprotective effects that support human health. Their intake may help prevent or mitigate chronic diseases linked to oxidative stress and inflammation. The extant literature suggests that they may have a role to play in the prevention and treatment of neurodevelopmental and neurodegenerative disorders. Further clinical and mechanistic studies are warranted.

## Figures and Tables

**Table 1:** Chemical classification of flavonoids [3,16].

Class	Skeletons Structure	Flavonoid	Source
Flavones	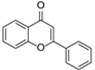	- Chrysin, - Apigenin - Luteolin	- *Parsley* - *Celery* - *Chamomile*
Flavonols	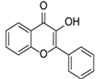	- Rtin - Querectin - Morin	- Onions - Kale - Broccoli
Flavanones	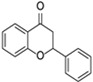	- Liquiritin - Hesperetin - Butin	- Oranges - Grapefruits - Lemons
Flavanols	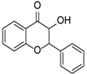	- Quercetin - Myricetin - Tamarixetin	- Pears - *Fava beans* - *Cinnamon*
Flavan-3-ols	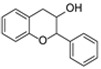	- Catechin - Epicatechin galate - Thearubigin	- Pears - Cherries - *Fava beans*
Anthocyanidins	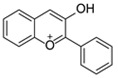	- Apigenidin, - Cyanidin - Epicatechin gallate	- Blueberries - Purple carrots - Black rice
Chalcones	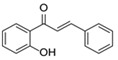	- Xanthoangelol - Mornigrol - Kurarinone	- *Ashitaba* - *White mulberry* - *Sophora flavescens*
Isoflavones	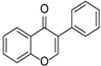	- Soy isoflavones - Glycitein - Daidzein	- *Chickpea* - Soybean - *Red clover*
Isoflavonones	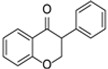	- Rotenone - Santalone - Equol	- *Derris* - *Lonchocarpus* - *Dalbergia odorifera*
Flavan-3,4-diols	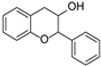	- Leucocyanidin - Leucodelphinidin - Leucofisetinidin	- *Anadenanthera peregrina* - *Nepenthes* - *Acacia maidenii*
Aurones	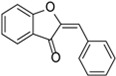	- Snapdragon - Cosmos - Dahlia	- *Antirrhinum majus* - *Cosmos* spp. - *Dahlia* spp.
Xanthones	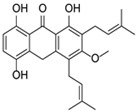	- Oxygenated xanthones - Prenylated xanthones - Bxanthones	- *Acanthaceae* - Fungi, - *Lichens*
Biflavonoids	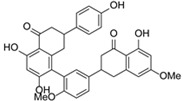	- Linobiflavnoid - Acuminatanol - Kolaviron	- *Thym* - Anac - *Garcinia kola*
Homoisoflavones	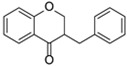	- Brazilin - Sappanol - Mimosin	- *Caesalpinia sappan* - *Agave tequilana* - *Ledebouria revoluta*

**Table 2:** The most important enzymes involved in the flavonoid biosynthesis pathways [35,47,48].

Pathway	Key Enzymes	Enzymes Main Function
Shikimate/ Phenylpropanoid	1. PAL	1. Converts phenylalanine to cinnamic acid
	2. C4H	2. Hydroxylates cinnamic acid to p-coumaric acid
	3. 4CL	3. Activates p-coumaric acid to p-coumaroyl-CoA
Polyketide/ Chalcone	1. CHS	1. Condenses p-coumaroyl-CoA with malonyl-CoA units to chalcone
	2. CHI	2. Converts chalcone to flavanone
	3. F3H	3. Hydroxylates flavanones to dihydroflavonols
	4. FLS	4. Converts dihydroflavonols to flavonols
	5. OMT	5. Adds methyl groups to hydroxyl groups on flavonoids
	6. UGT	6. Adds sugar moieties to flavonoids
	7. PKS	7. Catalyzes formation of chalcone in polyketide pathway

**Table 3:** Selected foods and their flavonoid contents (mg 100 g^−1^ edible portion) [58].

Food Item	Flavonoid Subclass	Representative Flavonoid	Mean Content (mg 100 g^−1^)
Red onion, raw	Flavonols	Quercetin	39.21
Grapefruit, raw	Flavanones	Naringenin	53
Grapefruit, raw	Flavanones	Hesperetin	1.5
Grapefruit, raw	Flavonols	Kaempferol	0.4
Red onion, raw	Flavones	Apigenin	0.24
Red onion, raw	Flavones	Luteolin	0.16
Red onion, raw	Flavonols	Myricetin	2.16
Cacao beans	Flavan-3-ols	(+)-Catechin	88.45
Cacao beans	Flavan-3-ols	(−)-Epicatechin	99.18
Tea, green, brewed	Flavan-3-ols	EGCG	26.05
Tea, black, brewed	Flavan-3-ols	(+)-Catechin	1.51
Blueberries, raw	Anthocyanidins	Pelargonidin	45.51
Strawberries, raw	Anthocyanidins	Cyanidin	32
Broccoli, cooked	Flavonols	Myricetin	2.6
Parsley, raw	Flavonols	Kaempferol	12.3

**Table 4:** Major flavonoid subclasses, their dietary sources, health benefits, and mechanisms of action [49,50,53,56,65,67].

Flavonoid Subclass	Representative Sources	Potential Health Benefits	Key Mechanisms of Action
Flavan-3-ols	Tea (green, black), cocoa, apples, grapes	Cardiovascular health improvement; enhanced insulin sensitivity	Antioxidant activity, improved endothelial function, lipid modulation, blood pressure reduction
Anthocyanidins	Berries, cherries, red cabbage, berry fruits	Neuroprotection; reduced risk of cardiovascular disease	Anti-inflammatory, antioxidant, support of cognitive function
Flavanones	Citrus fruits (oranges, grapefruits), orange juice, limes	Cardiovascular protection, anti-diabetic effects	Lipid regulation, improved insulin sensitivity
Flavonols	Onion, broccoli, parsley, apples	Cancer prevention, neuroprotection, reduced mortality	Apoptosis modulation, anti-inflammatory, antioxidant
Flavones	Parsley, celery, thyme	Anti-inflammatory, anticancer	Cellular signaling modulation, antioxidant

**Table 5:** Classification of flavonoid signaling pathways in the context of neurodevelopmental and neurodegenerative disorders [92,93].

Biological Function	Example Signaling Pathways	Role in Neurodevelopmental/Neurodegenerative Disorders
Anti-inflammatory	NF-κB, STAT3, MAPK (p38, JNK)	Reduction of neuroinflammatory processes in Alzheimer’s and Parkinson’s
Antioxidant	Nrf2/ARE, Keap1–Nrf2	Protection against oxidative stress in neurons
Cell survival/proliferation	PI3K/Akt/mTOR, Wnt/β-catenin	Supporting neuron survival and neurogenesis
Apoptosis-related	MAPK/ERK, p53, caspase-dependent pathways	Inhibition of excessive neuronal apoptosis
Oxidative stress	Nrf2/ARE, Keap1–Nrf2, SOD, CAT, GPx, ROS	Protection from ROS-induced neuronal damage
Mitochondrial function	AMPK, PGC-1α, mitochondrial biogenesis, ROS	Limiting mitochondrial dysfunction in aging and NDD
Gut–Brain Axis regulation	Microbiota, SCFAs, vagus, tryptophan–kynurenine	Microbiota-immune-brain interactions

## Data Availability

The data presented in this study are available on request from the corresponding author.
